# Characteristics of the pediatric population with gender incongruence attending specialized care in Cali, Colombia: an observational, descriptive and retrospective study

**DOI:** 10.1186/s13034-023-00689-6

**Published:** 2024-01-03

**Authors:** Kenny Gutiérrez, Mabel Moreno, Jimena Alexandra Sierra, Rodrigo Lemus, Karen Apraez, Mario Jr. Angulo

**Affiliations:** 1 Servicio de Endocrinología pediátrica, Fundación Valle del Lili, Cra 98 No.18-49, 760032 Cali, Colombia; 2 Centro de Investigaciones Clínicas (CIC), Fundación Valle del Lili, Cra 98 No.18-49, 760032 Cali, Colombia; 3 Universidad Icesi, Facultad de ciencias de la salud, Cl. 18 #122-135, Barrio Pance, 760032 Cali, Colombia

**Keywords:** Gender incongruence, Children, Adolescent, Transgender

## Abstract

**Background:**

Gender incongruence can often manifest itself from early childhood [Olson KR, Gülgöz S. Child Dev Perspect. 2018;12:93–7. https://doi.org/10.1111/cdep.12268] with a significant psychological impact, altering social and school dynamics without the appropriate care.[Tordoff DM, et al. JAMA Netw Open. 2022;5(2): e220978. https://doi.org/10.1001/jamanetworkopen.2022.0978] Early identification and gender–affirming care are essential to reduce adverse mental health outcomes, such as depression and self-harm [Tordoff DM, et al. JAMA Netw Open. 2022;5(2): e220978. https://doi.org/10.1001/jamanetworkopen.2022.0978].^.^This study aims to analyze characteristics and to estimate relative frequencies of gender incongruence in a population of children and adolescents receiving gender-affirming care at a high-complexity university hospital located in the third largest city in Colombia.

**Methods:**

This was a retrospective descriptive study of patients under 18 with gender incongruence that received gender-affirming care between January 2018 and June 2022 at Fundacion Valle del Lili in Cali, Colombia. Sociodemographic and clinical characteristics of 43 patients were assessed, as well as the relative frequencies of gender incongruence. Data analysis was performed with the statistical package STATA®. To determine significant differences between the characteristics of the patients who participated in the study, the Mann‒Whitney U test was performed for numerical variables with non-parametric distribution, while either Pearson's Chi-2 test or Fisher's exact test was performed for categorical variables.

**Results:**

For every ten individuals assigned female at birth, who manifested gender incongruence, there were eight assigned male at birth. The median age of onset of gender incongruence was ten years (IQR: 5–13 years), and the median time elapsed between the reported onset of gender incongruence and the first consultation with a multidisciplinary gender-affirming team was three years (IQR: 1–10 years).

The frequency of transgender identity was notable in participants with ages between 15 and 17 years. Depressive symptoms, anxiety, and psychotropic drug use were significantly higher in individuals assigned female at birth. Among 25 individuals assigned female at birth who participated in this study, 60% self-recognized as transgender men.18 individuals assigned male at birth, 67% self-recognized as transgender women. The most frequent treatment was a referral to mental health services (46.51%).

**Conclusion:**

Based on the cohort of our study, we can conclude that patients consult for gender-affirming treatment 3 years after the onset of gender incongruence. Anxiety and depression were higher in individuals assigned female at birth. Additionally, they presented at a later stage of sexual maturation, reducing the possibility of using puberty blockers.

## Background

Gender incongruence is defined as a condition in which the gender identity of an individual does not line up with the gender assigned at birth [1–3]. Psychological, familial, occupational, and social concerns often arise with experiences of gender incongruence due to conflicts with established cultural expectations for the assigned sex [4]. Some studies indicate that gender identity develops between the ages of three and five years [2–5] but many transgender and non-binary youth begin experiencing gender incongruence at or around puberty [3].^.^ However, some individuals may exhibit diverse gender expressions from childhood as an expected aspect of human developmental behavior, which may not necessarily reflect gender incongruence or transgender identity. There are few epidemiological studies of gender incongruence in childhood [4, 6, 7]. The first studies published in the 1960s reported the prevalence of transgender men to be 1/100,000 and transgender women to be 1/400,000. Later studies have shown a higher prevalence, especially in the European population, with values of 1/11,900 for transgender men individuals and 1/30,400 for transgender women individuals [5–8, 8, 9, 9–12]. Currently, there are no studies describing the sociodemographic, clinical characteristics and the incidence or prevalence of gender incongruence in the childhood population of Colombia [10, 13].

Knowledge of the baseline characteristics and trends in gender identity in this pediatric population helps to formulate specific care strategies and reduce poor outcomes in certain health indicators, such as, depression, anxiety, suicide and self-destructive behaviours [2, 14].

Given the scarcity of studies in developing countries that examine the psychological and emotional impact of gender identity incongruence in children and adolescents, this study was designed to carry out a sociodemographic characterization and estimate the frequency of gender incongruence in children and adolescents seeking gender-affirming care in a high complexity university hospital in the third largest city in Colombia.

## Materials and methods

### Study design

This was an observational, descriptive, and retrospective study based on the review of electronic medical records of patients under 18 with gender incongruence (ICD-10 code: F64-F642-F648-F649-F668) who received gender-affirming care between January 1, 2018, and June 30, 2022, at the Hospital Universitario Fundación Valle del Lili in Cali, Colombia. “Gender incongruence” is included in the new International Classification of Diseases ICD-11, and the objective of the condition is not to improve or eliminate symptoms but to facilitate gender-affirming care [10, 15]. However, in the current study, we used the international ICD-10 classification because this classification is still in effect within the institution.

#### Data collection procedures and techniques

Medical records of those individuals seeking gender-affirming care diagnosed with cognitive deficits and those containing incomplete sociodemographic and clinical information were excluded from the study. A database was created in BD Clinic to record the information. The analysis included the following sociodemographic variables: age in years during the first specialized consultation, age in years at the reported onset of gender incongruence, years elapsed since the first assessment, nationality, and socioeconomic level according to the DANE (Colombia National Administrative Department of Statistics), based on the conditions of the housing in which the family group resides and the environment or area in which the housing is located. For this reason, the socioeconomic level is classified into: Stratum 1 (Low-low), stratum 2 (Low), stratum 3 (Lower-middle), stratum 4 (Middle), stratum 5 (Upper-middle) and stratum 6 (High).

Common comorbidities, such as anxiety, depression, self-harm, suicide attempt, use of psychotropic drugs, family history of depression and anxiety, autism spectrum disorder (ASD), and use of psychoactive substances, were also included. Additionally, the individual’s Tanner scale stage at the first assessment and the type of gender-affirmative treatment received were recorded. Of the 46 individuals who sought care at the gender clinic during the study period, 3 were excluded for not meeting the selection criteria. Thus, 43 patients were enrolled in the study.

### Statistical analysis

Data analysis was performed with the statistical package STATA® version 16.0. Initially, the normality of the quantitative variables was evaluated with the Shapiro‒Wilks test. The median and interquartile range were calculated for numerical variables with a nonparametric distribution, and the absolute and relative frequencies were calculated for categorical variables. To determine significant differences between the characteristics of the women and men who participated in the study, the Mann‒Whitney U test was performed for numerical variables, while either Pearson's Chi-2 test or Fisher's exact test was performed for categorical variables. For all tests, the level of statistical significance was defined as a p value < 0.05. The prevalence of gender incongruence was estimated with 95% confidence intervals for different sociodemographic categories including the sex assigned at birth, age group, and presence of a pathological or pharmacological history.

## Results

### Sample characteristics

Between 2018 and 2022, 43 participants between the ages of 3 and 17 were evaluated in the gender clinic. 58% were assigned female at birth and 42% were assigned a male at birth. Half of the individuals were younger than 15 at the first assessment, with no statistically significant differences found between the two biological sexes. For every ten individuals assigned female at birth who manifested gender incongruence, there were eight assigned male at birth. The median age of onset of gender incongruence, was 10 years for the total sample (IQR: 5–13 years), with 11 years for half of the females (IQR: 6–13 years) and 5 years for half of the males (IQR: 4–13 years). The time between incongruence onset and first admission to the gender clinic was 3 years (IQR: 1–10 years). Most of the subjects analyzed were born in Colombia, and more than half came from families with a middle socioeconomic level (strata 3 and 4) (Table 1). The occurrence of depression, anxiety, and the use of psychotropic drugs was statistically more significant in individuals assigned female at birth. Other behaviors, such as self-injury, suicide attempts, family history of depression and anxiety, and the use of psychoactive substances, were similar in both sexes.

**Table 1:** Characteristics of pediatric patients with gender identity incongruence

	General, n = 43 (100%)	Sex assigned at birth		
		Women^b^, n = 25 (58.14%)	Men ^b^, n = 18 (41.86%)	p value
Characteristic				
Age in years at first consultation^a^	15 (13–16)	15 (13–17)	15 (7–16)	0.388^c^
Age in years at the reported onset of gender nonconformity^a^	10 (5–3)	11 (6–13)	5 (4–13)	0.095^c^
Years elapsed for the first evaluation^a^	3 (1–10)	3 (1–10)	3 (2–8)	0.930 ^c^
Nationality n (%)				
Colombia	40 (93.02)	23 (92)	17 (94.44)	0.624^d^
Another country	3 (6.98)	2 (8)	1 (5.56)	
Socioeconomic status n (%)				
Stratum 2	5 (11.63)	2 (8)	3 (16.67)	0.173^d^
Stratum 3	11 (25.58)	5 (20)	6 (33.33)	
Stratum 4	11 (25.58)	8 (32)	3 (16.67)	
Stratum 5	7 (16.28)	6 (24)	1 (5.56)	
Stratum 6	3 (6.98)		3 (16.67)	
No data	6 (13.95)	4 (16)	2 (11.11)	
Depression. n (%)				
No	24 (55.81)	9 (36)	15 (83.33)	0.002^e^
Yes	19 (44.19)	16 (64)	3 (16.67)	
Anxiety n (%)				
No	27 (62.79)	10 (40)	17 (94.44)	0.000 ^e^
Yes	16 (37.21)	15 (60)	1 (5.56)	
Self-harm n (%)				
No	32 (74.42)	16 (64)	16 (88.89)	0.094 ^b^
Yes	10 (23.26)	8 (32)	2 (11.11)	
No data/did not report	1 (2.33)	1 (4)		
Suicide attempt n (%)				
No	35 (81.40)	19 (76)	16 (88.89)	0.344^d^
Yes	7 (16.28)	5 (20)	2 (11.11)	
No data/did not report	1 (2.33)	1 (4)		
Use of psychoactive drugs, n (%)				
No	22 (51.16)	9 (36)	13 (72.22)	0.040^d^
Yes	21 (48.84)	16 (64)	5 (27.78)	
Family history of depression and anxiety, n (%)				
No	18 (41.86)	10 (40)	8 (44.44)	0.284 ^e^
Yes	13 (30.23)	8 (32)	5 (27.78)	
No data/ Did not report	12 (27.91)	7 (28)	5 (27.78)	
Autism Spectrum Disorder, n (%)				
No	30 (85.71)	17 (68)	13 (72.22)	0.321 ^d^
Yes	5 (69.77)	4 (16)	1 (5.56)	
No data/ Did not report	8 (18.60)	4 (16)	4 (22.22)	
Use of psychoactive substances, n (%)				
No	26 (60.47)	12 (48)	14 (77.78)	0.468 ^d^
Yes	5 (11.63)	3 (12)	2 (11.11)	
No data/ Did not report	12 (27.91)	10 (40)	2 (11.11)	
Tanner scale at first consultation, n (%)				
Stage I	6 (13.95)		6 (33.33)	
Stage II	2 (4.65)	1 (4)	1 (5.56)	0.000 ^d^
Stage III	5 (11.63)	1 (4)	4 (22.22)	
Stage IV	2 (4.65)		2 (11.11)	
Stage V	27 (62.79)	22 (88)	5 (27.78)	
No data/ Did not report	1 (2.33)	1 (4)		
Treatment, n (%)				
Mental health intervention	20 (46.51)	13 (52)	7 (38.39)	0.003 ^d^
Puberty Blockers—GnRH analogs	10 (23.26)	5 (20)	5 (27.78)	
Cross-hormone therapy with testosterone	7 (16.28)	7 (28)		
Cross-hormone therapy with estrogen	4 (9.30)		4 (22.22)	
No data/did not report	2 (4.65)		2 (11.11)	

^a^Median (IQR: interquartile range); ^b^Sex assigned at birth; ^c^Mann‒Whitney U test; p value < 0.05 statistically significant; ^d^Fisher's exact test: p value < 0.05 statistically significant: ^e^Chi-2 test; p value < 0.05 statistically significant

The Tanner scale performed during the first consultation showed that 63% of the participants were classified as stage V, that is, with external physical and sexual characteristics (breasts, testicular volume, and pubic hair development) that correspond to complete sexual development. Breaking it down by birth assigned sex, 88% assigned female at birth and 28% assigned male at birth in this study were classified as Tanner stage V, with statistical significance between both groups. Regarding the type of treatment provided, significant differences were found in both sexes; a high percentage assigned female at birth received mental health treatment while a high percentage assigned male at birth received treatment with GnRH analogs (Table 1). The trend in the use of GnRH as hormonal treatment in individuals assigned male at birth is related with earlier Tanner stages observed during the initial evaluation when the level of sexual characteristic maturation is still incipient (Tanner I, II, III). In contrast, treatment by mental health was more common among individuals assigned female at birth, as most of these patients presented for the initial assessment with complete sexual maturation (Tanner V).

### Gender incongruence

Of the 25 individuals assigned female at birth who participated in the study, 60% (CI95% = 40.69–76.63) self-recognized as transgender men, and 32% (CI95% = 17.06–51.73) reported being in the exploratory phase. Of the 18 participants assigned male at birth, 67% (CI95% = 43.57–83.89) self-recognized as transgender women and 33% (CI95% = 16.10–56.42) reported being in the exploratory phase. The highest percentage of gender incongruence occurred in participants between 15 and 17 years of age (60%), of which 42% of this subgroup identified as transgender men (CI95% = 25.52–61.07) and 34% as transgender women (CI95% = 19.31–53.87) (Table 2).

**Table 2:** Characteristics of pediatric patients with gender identity incongruence

Characteristics	Trans male		Trans female		Non-binary		Exploratory phase		Total	
	n (%)	95% + CI	n (%)	95% + CI	n (%)	95% + CI	n (%)	95% + CI	n (%)	95% + CI
Sex assigned at birth*										
Female	15 (60)	40.69–76.63	–	–	2 (8)	10.86–26.10	8 (32)	17.06–51.73	25 (58.14)	42.12–72.98
Male	–	–	12 (66.67)	43.57–83.89	–	–	6 (33.33)	16.10–56.42	18 (41.86)	27.01–57.87
Age groups										
0–4 years	–	–	–	–	–	–	3 (100)		3 (6.98)	14.62–19.06
5–9 years	–	–	2 (66.67)	20.24–94.37	–	–	1 (33.33)	5.62–79.75	3 (6.98)	14.62–19.06
10–14 years	4 (36.36)	14.98–64.80	1 (9.09)	0.00–39.90	2 (18.18)	3.98–48.84	4 (36.36)	14.98–64.80	11 (25.58)	13.51–41.17
15–17 years	11 (42.31)	25.52–61.07	9 (34.62)	19.31–53.87	–	–	6 (23.07)	10.70–42.38	26 (60.46)	44.40–75.02
Depression										
No	4 (16.67)	4.73–37.38	10 (41.67)	22.10–63.35	1 (4.17)	0.1–21.12	9 (37.50)	18.79–59.40	24 (55.81)	39.87–70.92
Yes	11 (57.89)	33.49–79.74	2 (10.52)	13.01–33.13	1 (5.26)	1.3–26.02	5 (26.32)	9.14–51.20	19 (44.18)	29.07–60.12
Anxiety										
No	6 (22.22)	8.62–42.25	11 (40.74)	22.38–61.20	1 (3.70)	0.0–18.79	9 (33.33)	16.51–53.96	27 (62.79)	46.72–77.02
Yes	9 (56.25)	29.87–80.24	1 (6.25)	1.58–30.23	1 (6.25)	1.58–30.23	5 (31.25)	11.01–58.66	16 (37.21)	22.97–53.27
Use of psychotropic drugs										
No	5 (22.72)	7.82–45.37	8 (36.36)	17.19–59.34	–	–	9 (40.90)	20.70–63.64	22 (51.16)	35.46–66.69
Yes	10 (47.62)	25.71–70.21	4 (19.05)	5.44–41.90	2 (9.52)	1.17–30.37	5 (23.81)	8.21–47.16	21 (48.83)	33.30–64.53

*Sex assigned at birth

^+^ 95% CI: 95% confidence intervals

Among patients with a history of depression, anxiety, and psychotropic drug use, nearly half or more than half identified as transgender men (Table 2).

## Discussion

This retrospective descriptive study is the first to provide demographic and clinical data and the frequency of gender incongruence in a sample of children and adolescents evaluated in the gender clinic of a university hospital in Cali, Colombia. Of the 43 patients, 58% were assigned female at birth and 42% were assigned male at birth. This higher representation assigned female at birth has also been reported in other studies [15, 16]. This trend is thought to be because assigned female at birth with gender incongruence are more socially accepted than those assigned male at birth. [15, 16].

The results of the study show that gender incongruence is present in the pediatric population, with a higher percentage of patients assigned male at birth identifying as transgender women (67%) and assigned female at birth identifying as transgender men (60%). This data is consistent with reports from other studies where the prevalence of transgender identity in assigned men at birth tends to be higher than those assigned female at birth. [7, 11–14, 18–21].

Age was a determining factor in the identification of gender incongruence in this cohort. Subjects between 15 and 17 years of age more frequently expressed gender incongruence compared to patients between 10 and 14 years of age. A similar trend was observed in an analytical longitudinal study conducted in the Netherlands, in which 37% of children who expressed gender incongruence between 5 and 17 years of age continued to endorse a sense of gender nonconformity after several years of follow-up [17, 22]. Although our data do not include the follow-up overtime necessary to assess the persistence of gender incongruence from younger ages, it allowed us to identify, with clinical criteria, the age groups where many adolescents recognize their gender identity.

The evolution of gender incongruence is a topic undergoing investigation. Different prospective observational studies have been carried out to evaluate the persistence of gender incongruence in children and adolescents into adulthood, and these persistence rates ranged from 27 to 81% [28]. Factors associated with persistence are still under investigation, but some that have been identified include higher intensities of gender dysphoria and more body dissatisfaction [28].

The early onset of gender incongruence in our study population is another significant finding. Half of the participants began to experience gender incongruence approximately at age ten years, with five years of age being the earliest onset found in those assigned males at birth. The time elapsed between onset of incongruence and the first consultation or first care by a multidisciplinary team of experts was three years for all patients. Half of the subjects came to the first consultation when physical pubertal stage development was advanced. Gender incongruence tended to be more defined in this stage, especially for those assigned females at birth.

A significantly higher frequency of anxiety, depression, and psychotropic drug use was—those who identified as transgender men. Different studies have reported higher rates of psychiatric comorbidities in gender-nonconforming pediatric populations [20, 21, 23, 24], including anxiety, depression [2, 5], and suicidal ideation [22, 25]. A study conducted in Chicago [23, 26] revealed significantly lower rates of depression in transgender women than in transgender men. The results show that people assigned female at birth are more likely to suffer emotional distress, due to social expectations and gender inequality.

It is essential to mention that since its inception, the gender clinic at Fundación Valle del Lili has implemented a multidisciplinary approach to patient care, with a team composed of mental health, endocrinology, and primary care physicians who collaborate to assess and establish an accurate diagnosis, make decisions and provide individual recommendations regarding treatment according to age and stage of puberty (prepubertal, mid-pubertal and postpubertal [24–27]. For all stages, the primary treatment for participants with gender nonconformity was mental health intervention that aimed to help the minor and his or her family navigate identity exploration and acceptance, identify possible psychiatric comorbidities, provide strategies to help strengthen integration into their environment, and minimize risk behaviors based on the recommendations provided by WPATH and the gender affirmative model [28, 29].

## Study limitations

One limitation of the study is the sample size, which makes it difficult to generalize the results. It is essential to know the sociodemographic and clinical profile of children and adolescents with gender incongruence as it will allow us to strengthen health care and treatment in the future, according to the characteristics and needs of our pediatric population.

## Conclusion

Based on the cohort of our study, we can conclude that patients consult for gender-affirming treatment 3 years after the onset of gender incongruence. However, we are unaware of the reasons for the delay in receiving gender-affirming treatment. Additionally, individuals assigned female at birth present higher percentage of anxiety and depression, also arrived with a complete level of sexual maturation, which reduces the possibility of using puberty blockers.

## Data Availability

The data that support the findings of this study are available from Fundación Valle del Lili but restrictions apply to the availability of these data, which were used under license for the current study, and so are not publicly available. Data is however available from the authors upon reasonable request and with permission of Fundación Valle del Lili.
